# To share or not to share: communication of caregiver-reported outcomes when a patient has colorectal cancer

**DOI:** 10.1186/s41687-022-00418-1

**Published:** 2022-02-05

**Authors:** A. Fuchsia Howard, María-José Torrejón, Kelsey Lynch, Scott M. Beck, Sally Thorne, Leah Lambert, Antony Porcino, Mary A. De Vera, Janine M. Davies, Jonathan Avery, Angela C. Wolff, Melanie McDonald, Joyce W. K. Lee, Penelope Hedges, Mary T. Kelly, Michael McKenzie

**Affiliations:** 1 School of Nursing, The University of British Columbia, T201-2211 Wesbrook Mall, , Vancouver, BC V6T 2B5 Canada; 2 BC Cancer, Vancouver, BC V5Z 4C2 Canada; 3 Faculty of Pharmaceutical Sciences, The University of British Columbia, Vancouver, BC V6T 1Z3 Canada; 4 Faculty of Medicine, The University of British Columbia, Vancouver, BC V6T 1Z3 Canada; 5 School of Nursing, Trinity Western University, Langley, BC V2Y 1Y1 Canada

**Keywords:** Caregiver, Family, Oncology, Colorectal cancer, Caregiver-reported outcomes, Measurement, Supportive care, Psychosocial, Qualitative research, Interpretive description

## Abstract

**Background:**

The importance of patient-centered measurement in cancer care has led to recognition of the potential for caregiver-reported outcomes to improve caregiver, patient and healthcare system outcomes. Yet, there is limited evidence to inform caregiver-reported outcome implementation. Our purpose was to generate evidence to inform the meaningful and constructive integration of caregiver-reported outcomes into cancer care to benefit caregivers, including exploration of the question of the extent to which these assessments should be shared with patients. We focused on caregivers of patients with colorectal cancer (CRC) because CRC is common, and associated caregiving can be complex.

**Results:**

From our Interpretive Description analysis of qualitative interview data from 78 participants (25 caregivers, 37 patients, and 16 healthcare providers [HCPs]), we identified contrasting perspectives about the sharing of caregiver-reported outcome assessments with patients with CRC. Those who preferred open communication with both the patient and caregiver present considered this essential for supporting the caregiver. The participants who preferred private communication without the patient, cited concern about caregiver- and patient-burden and guilt. Recognizing these perspectives, HCPs described strategies used to navigate sensitivities inherent in preferences for open versus private communication.

**Conclusions:**

The integration of caregiver-reported outcomes into cancer care will require careful consideration of caregiver and patient preferences regarding the communication of caregiver assessments to prevent additional burden.

## Introduction

Primary informal caregivers of patients with cancer, hereafter referred to as caregivers, are increasingly recognized as instrumental partners in healthcare. Among Americans treated for cancer in the prior 3 years, 55% reported having an informal caregiver [1]. Caregivers are key family members or friends who provide emotional and functional support throughout the cancer trajectory, including managing patients’ symptoms and side effects, helping patients cope with emotional distress, assisting with daily living, and coordinating with healthcare providers (HCPs) [2]. Despite their best efforts to manage these demands, inadvertently exceeded capabilities may result in high physical, emotional, social, spiritual, and financial burden – collectively termed caregiver burden [3]. In an American study, 62% of cancer caregivers were in a high burden situation and averaged 33 hours a week providing care, with 32% providing 41 or more hours of care weekly – the equivalent of a full-time job [4]. Considering the enormity of caregiving, it is not surprising that caregivers experience psychological distress and poor mental and physical health [5], with some research suggesting that higher caregiver burden is associated with increased risk of caregiver morbidity and mortality [6, 7]. Yet, caregiver needs are frequently overlooked [8]. Moreover, assessment of caregiver outcomes is not standard of care in many oncology settings, nor is the offering of caregiver support. This is a remarkable gap in care considering that the unmet needs of caregivers might rival those of patients [8].

Evidence suggests that use of patient-reported outcome measures in cancer care can improve patient outcomes, quality of life, survival, and health system outcomes [9–15]. With growing recognition of family-centered care, a focus on caregiver-reported outcomes is also emerging. Caregiver-reported outcomes refer to a caregiver’s assessment of their own health status and health-related quality of life as a result of caring for a patient with cancer. A recent international study highlighted the need to develop means of identifying caregivers at highest risk for burden and integrating caregiver assessments into standard care [16]. In 2020, the Canadian Patient-Reported Outcomes National Steering Committee similarly called for the systematic integration of caregiver assessments [17]. Preliminary studies suggest that eliciting caregiver-reported outcomes is feasible [18, 19], yet evidence is necessary to inform their implementation in cancer care.

Our overarching research objective was to identify what caregiver-reported outcomes are important to caregivers and how to integrate this measurement into cancer care to meet caregivers’ needs. This research was centered on caregivers of patients with colorectal cancer (CRC) because, in Canada, CRC is the third most commonly diagnosed cancer [20] and is associated with demanding caregiving given the complexity and involvement of treatments such as surgery requiring an ostomy, chemotherapy, and radiotherapy [2, 5]. The analysis we report on here specifically focuses on one aspect of the integration of caregiver-reported outcomes into cancer care, the sharing of these assessments with the patient with CRC, from the perspective of caregivers, patients with CRC, and HCPs.

## Methods

This qualitative, Interpretive Description [21], patient-oriented research [22] was conducted virtually owing to the COVID-19 pandemic. This was a change from the initially planned data collection that was primarily going to consist of in-person interviews with study participants. Patient- and caregiver-partners (individuals with lived experience), clinicians, and multidisciplinary stakeholders were equal team members throughout the research [22]. The protocol was approved by the harmonized University of British Columbia, BC Cancer Research Ethics Board. Further details of study methods have been published elsewhere [23].

### Setting and sample recruitment

We conducted this research in British Columbia, Canada, where there is a publicly funded healthcare system serving a population exceeding 5 million. Caregiver-reported outcome assessments are not part of standard care for patients with CRC. Caregiver participants were ≥ 19 years of age, spoke English, and involved in providing care to a relative (a spouse, unmarried partner, parent, sibling, adult child), a neighbour, or a friend with CRC. Patient participants were diagnosed with CRC, ≥ 19 years of age, and spoke English. Study information was distributed via the online newsletters and social media pages of caregiver and oncology organizations, and through an email listserv of individuals that consented to be contacted for research purposes. Eligible HCPs, including those whose current practice included care of CRC patients either in acute or community settings were identified through the research team’s professional networks and emailed an invitation to participate. All individuals who contacted the research team, who met eligibility criteria and agreed to participate were included. All individuals who contacted the research team spoke English and so no one was excluded on this basis. We had intended to purposefully recruit participants through oncology and community healthcare settings to obtain a sample of individuals who represented diversity in participant characteristics and experiences. However, this purposive sampling was not possible owing to the COVID-19-related restrictions and our online recruitment, and we were reliant on a convenience approach to sampling. Thus, we included more participants than initially planned in our aim for maximal variation in the sample.

### Data collection

We conducted in-depth, semi-structured participant interviews virtually via Zoom from 04/2020 to 11/2020 and offered caregiver and patient participant pairs the option of doing interviews separately or together as a dyad (caregiver and patient interviewed together). Interview guides included questions about priorities for caregiver-reported outcome assessments, the communication of these assessments to HCPs and the patient, and recommendations for the implementation of caregiver-reported outcomes into routine practice. Interviews lasted 30–90 min, were audio recorded, transcribed verbatim, de-identified, and checked for accuracy. Rather than aiming for theoretical saturation, we focused on gathering interview data that was high in information power [24]. Information power was enhanced by interviewers possessing qualitative interviewing knowledge and skills and all study participants having firsthand experiences relevant to the research aims with wide variation in their in-depth accounts.

### Data analysis

Using an Interpretive Description approach [21] and the data management software NVivo™ Version 12, we identified all data relevant to assessment of caregiver-reported outcomes. Next, we inductively identified patterns, diversities, and initial categories within the assessment of caregiver-reported outcomes data, which informed our development and application of a coding frame to the data by three coders. One team member then compared and contrasted pieces of data within and across participants, a technique known as constant comparison [25]. We conducted the initial analysis on the caregiver and patient interview data, analyzing the caregiver and patient data simultaneously, and then compared the developing findings with the HCP interview data. This resulted in the grouping and regrouping of our analytic categories. As this analytic process continued, our research team engaged in ongoing deliberations, which informed subsequent refining of the emerging findings. Data analysis proceeded until we were confident that our conceptualization of the findings represented the considerations that were most important to participants specific to the sharing of caregiver-reported outcome assessments with the patient.

## Results

A total of 78 individuals (25 caregivers, 37 patients, and 16 HCPs) participated (see Table 1 for demographic details). HCPs included 6 nurses, 1 nurse practitioner, 2 family physicians, 1 medical oncologist, 1 radiation oncologist, 2 social workers, 2 registered dietitians, and 1 genetic counsellor.

**Table 1:** Caregiver and patient participant characteristics

	Caregiver (n = 25)	Patient (n = 37)
Characteristic	Number	Number
Mean age (years)	55	65
Gender		
Woman	22	16
Man	2	21
Non-binary	1	0
Relationship to the patient (you are the patient’s…)		
Husband/man partner	1	
Wife/woman partner	15	
Non-binary partner	1	
Daughter	6	
Son	1	
Friend (woman)	1	
Relationship to caregiver (you are your caregiver’s…)		
Husband/man partner		19
Wife/woman partner		8
Mother		1
Father		1
Sister		1
Daughter		1
Cousin (man)		1
Friend (woman)		2
Listed more than 1 type of relationship		3
Marital status		
Married/common-law/living together	20	27
Divorced/separated	1	4
Single	4	5
Widowed	0	1
Living arrangement		
Living with the patient or their caregiver	16	29
Living alone	5	7
Other	4	1
Employment status		
Full-time	8	6
Part-time	5	6
No	11	23
Other	1	2
Cancer stage of patient		
1	3	2
2	1	10
3	6	16
4	8	4
Unknown	7	5
Patient colostomy and/or ileostomy		
Yes	11	18
No	14	19

Our findings identify and describe contrasting perspectives among caregivers and patients about the sharing of caregiver-reported outcome assessments with the patient with CRC. While some caregivers preferred open and transparent communication about their caregiving experiences with the patient present, others preferred private communication without the patient. Complementing these findings, HCPs described positive and negative aspects of open versus private communication and contingent strategies they used to navigate sensitivities inherent in caregiver and patient preferences.

### Contrasting preferences among caregivers and patients: open versus private communication

#### Open communication

The willingness of caregivers and patients to have open and transparent discussions together with their HCPs about caregiver challenges was most evident among the 12 participants who had agreed to a dyad interview (a caregiver *with* a patient). That only 6 caregiver and patient pairs (12 participants) chose to take part in a dyad interview, demonstrates that most caregivers and patients were not comfortable communicating their views openly with each other present. The participants who did express a preference for openness and transparency tended to be couples and family members who self-identified with the practice of open communication and emotional sharing as a general feature of their relationship. A 74-year-old patient described how this capacity for open communication is a skill that he and his wife developed over the course of their marriage, and therefore, felt similarly comfortable doing so in the context of learning about potential caregiver struggles.

We have conversations that we don’t like having... It took a long time to get here, but we have them - that wouldn’t bother me. That’s why this [interview] is easy enough for us to do with you together. Would it bother other people? I’m sure. (Participant 30)

This patient continued to say that he expected most caregivers and patients would not voice honest opinions in the presence of each other unless they had developed the capacity for transparent communication and the ability to identify and express their own emotional experiences. Aligning with this dyad’s summation; few participants overall voiced an interest in a candid discussion.

Those who expressed a preference for open and transparent communication of caregiver challenges and caregiver-reported outcome assessments considered this essential for understanding, appreciating, and providing reciprocal support. There were numerous instances wherein patients conveyed their desire to know what their caregiver was experiencing, thereby enhancing the closeness of their relationship. This awareness was also considered essential to either supporting the caregiver or encouraging the caregiver to focus on self-care and seek additional informal or formal support. For example, a 58-year-old patient who stated he would have the capacity to respond to information that his non-binary caregiver was not doing well, added that he would instruct them *“to get some additional support”* (Participant 6). He framed his response with the caveat that they both might need to find additional support elsewhere to *“ease the burden on each [of us].”*

Despite these accounts, there also appeared qualifiers or limits to openness and transparency. A 56-year-old woman caregiver who characterized her friendship with the patient as *“pretty open,”* said it would be fine for him to know she faced difficulties as a caregiver. She said, *“if I’m stressed and trying to figure it all out, I would be open about that… I would just talk aloud. This is what we’re going to do”* (Participant 63). At the same time, she qualified her openness by explaining that *“the only thing I hid from him was my concern,”* referring to her concern about the speed of receiving medical interventions. Similarly, a 27-year-old caregiver (Participant 60) who described her relationship with her mother (patient) as *“very open,”* qualified her preference for transparent communication by clarifying that her mother was already acutely aware of the difficulty her illness imposed on the daughter and husband. She said her mother’s awareness of being a burden on others was more painful than the disease itself. In this case, open communication and emotional closeness in the family had its limits. The caregiver preferred to protect the patient from more distress that could be caused by additional open communication with HCPs about caregiver well-being or caregiver assessments.

#### Private communication

The majority of caregivers and patients (50 out of 62) in this study preferred a private interview without the patient present and, congruent with this choice, expressed a preference for private discussions with HCPs about caregiver challenges and caregiver-reported outcome assessments. Many caregivers were concerned that an open discussion of their challenges and struggles might worry the patient and provoke feelings of guilt. Thus, they did not feel it was possible to be fully honest in front of the patient. For example, a 40-year-old caregiver was asked how she expected her mother (patient) would respond to information that caregiving had its own struggles. *“I think she would have felt very guilty, and I just wouldn’t have told her”* (Participant 4). Caregivers exhibited a desire to protect the patient from any additional stress, preferring to shield them from knowledge that the ongoing care they needed could be difficult for the caregiver. A 60-year-old wife-caregiver outlined the tasks her husband’s illness had imposed on her life for years, but she said *“I don’t complain. I try not to even sigh heavily, [but] from time to time maybe it escapes”* (Participant 5). Many caregivers, immediately categorized discussions about their struggles as potential sources of stress for the patient and therefore required privacy. Another 72-year-old wife-caregiver expressed a similar view, wishing to protect her husband from any distress and would prefer a private assessment of her own health status:

I would probably [feel] more easy without him present, mostly because I wouldn’t want him to be worried about me when he was the source of the medical issues. I wouldn’t want him to worry about me. (Participant 62)

The patients also confirmed why it would be difficult, even distressing, to learn about their caregiver’s struggles. Some caregivers and patients were not used to discussions that involved emotional sharing. A 79-year-old patient recalled that he and his caregiver-daughter never talked about how his illness impacted her life because his attention was on his own health. *“I was just focusing on getting well, getting through this, so we didn’t have any discussions around what we were feeling”* (Participant 2). Other patients also expressed concern that if they learned their caregiver was experiencing challenges, this information could worsen their own precarious situation coping with cancer. These patients believed discussions about caregiver health and caregiver-reported outcomes were important but should not be done in front of them because, as one 74-year-old patient said when reflecting on her son and daughter as caregivers, *“when you’re a patient, you’re very vulnerable”* (Participant 7). Similarly, another 68-year-old patient commented firmly that a conversation between his caregiver-wife and a HCP should be held in private, because if he were to learn about any difficulties she faced, *“I think that would upset me, and I think it would upset a lot of people”* (Participant 24). The patient commentary throughout this study highlighted how caregiver-reported outcome assessments could threaten the identity of highly independent patients to the extent that they could feel unable to simultaneously focus on their own illness and the well-being of their caregiver.

### Healthcare provider perspectives: Open versus private communication and contingent strategies

#### Open communication

The HCPs considered that open and transparent communication with patients about caregiver challenges and caregiver-reported outcome assessments, either through a general discussion or with a measure, would help to ensure that patients’ needs were met; to ease patients' concerns about their caregiver; to reduce caregiver burden; and to support ethical care. In prioritizing patient well-being, HCPs commented that open communication made space for the patient to articulate whether and how caregiver challenges might be inadvertently affecting them and contributing to unmet patient needs. Similarly, some HCPs considered caregiver-reported outcome assessments to be helpful for easing patient worry about the caregiver *“taking this off the patient’s plate,”* reassuring the patient that the caregiver is being cared for, and freeing the patient up to focus on their own wellbeing. HCPs further portrayed open communication as a means to ease caregiver burden. One oncologist explained that while a caregiver might be ashamed and reticent to ask for assistance, a patient might be well-positioned to reciprocate support or help redistribute caregiver responsibilities:

The caregivers are providing a lot of care and if they’re struggling, maybe the patient could help play a role in perhaps trying to redistribute some of that burden on to maybe another child or something like that. Because that designated caregiver… they might feel a responsibility or an obligation to be that person. If they can’t, then maybe they don’t want to acknowledge it because they don’t want to disappoint the patient. But if the patient was made more aware that they’re struggling then it might make it easier to transition some of that burden to somebody else. (Participant 75)

Some HCPs considered open and transparent communication to be in alignment with ethical care, with an oncology nurse commenting that, *“it’s ethically problematic to be initiating care for the caregiver or initiating assessments for the caregiver without the patient’s knowledge”* (Participant 69), and a social worker endorsing transparency, *“because if all of a sudden someone doesn't know that they're being assessed, it can definitely bring up animosity. It can bring up, legal issues. It can affect trust” (*Participant 77).

Similar to caregiver and patient participants, the HCPs attributed the preference for open and transparent communication to the nature of the caregiver-patient relationship and their typical means of communication with each other. HCPs raised concerns, however, that this type of communication could inadvertently place additional strain on caregivers and patients *“who are already struggling a little bit in relationships”* or whose typical interactions do not include open discussions about their challenges. Moreover, qualifiers to open communication included connecting caregiver assessments to support and resources to prevent amplification of caregiver or patient guilt, as articulated by a family physician.

It’s not just about an assessment, it’s about an assessment in the context of having solutions and supports. Because if all you say is, well, we’re going to find out if this is going on and then that’s it. Don’t bother because then you’re just opening up a can of worms. But if it’s in the context of we’re doing this so that we can identify the needs and where we can try and help support your family, your caregivers, that’s a totally different message to the patient. (Participant 51)

#### Private communication

Echoing caregiver and patient concerns, HCPs were aware that patients often feel like a burden on their caregiver. HCPs pondered how the patient’s knowledge that their caregiver is struggling could exacerbate patient distress and guilt, as exemplified by one wound care/ostomy nurse.

Patients are already very concerned about the additional burden that they place on caregivers. I think caregivers don’t want to acknowledge or reinforce that in front of the patients. So, I don’t think that they feel the ability to be honest or open about their emotions or about the toll that it’s taking in front of the patient. They don’t want the patient to feel guilty in addition to having to deal with their disease. (Participant 68)

HCPs shared their observations that in their attempts to protect the patient, caregivers *“do not speak freely”* nor are they always honest about their struggles in front of the patient. According to HCPs, caregivers tend to be more willing and able to share their challenges without the patient present. Thus, HCPs characterized private communication as *“crucial, almost fundamental”* to supporting caregivers. Similar to how HCPs considered open communication as important for the ethical care of the patient, HCPs also cited private communication as supporting ethical care for the caregiver. One nurse practitioner contended that,

If the caregiver doesn’t want the patient to know there’s confidentiality in that too. I think that patients can be aware that an assessment is going to be going to happen. I don’t think that would be harmful, but the results of the assessment might be. (Participant 53)

Despite recognition that the option for private communication about caregiver challenges and assessments is vital, this rarely happens. Particularly in acute oncology care, the HCP rarely interacts with the caregiver without the patient present, which could prevent HCPs from assessing caregiver burden and proactively arranging supports and resources.

#### Healthcare provider contingent strategies to navigate interactions with caregivers and patients

Without standardized approaches for assessing caregiver-reported outcomes and their ethical concerns, the HCPs described the complexity they encountered when deciding if and how to have a conversation about caregivers’ challenges and needs. They overwhelmingly endorsed the need to individualize their approach to the preferences and needs of both the caregiver and the patient – that is, to support the caregiver’s choice to share information about their challenges and caregiver-reported outcome assessments and the patient’s choice to be made aware. Due to lack of an assessment tool or a specific guideline, some HCPs relied on their accumulated personal and professional wisdom to develop strategies that were contingent on caregivers’ and patients’ preferences for open versus private conversations. For this, some HCPs mentioned that they were attentive to caregivers’ and patients’ responses and non-verbal communication, as shared by a general practitioner:

Often in front of the patient, they'll [caregiver] say, “Oh, everything's fine, I'm fine”. But you look in the eyes and you know, it's not fine. And so you can get hold of them privately without the patient. Then you may get more of the truth.

An oncology nurse further indicated how the trust that patients have in HCPs gives them some leeway for *“overstepping”* to offer additional supports needed by the caregiver but unacknowledged by the patient, stating *“it's always leaving space, you know, ‘if you don't want it today, let us know because you're going to come back next week’”.* HCPs described using *“gentle probing questions”* and looking for opportunities to see how the caregiver is doing and to query deeper if they get the sense they are struggling. The HCPs also created opportunities to privately *“check-in”* with the caregiver by encouraging the patient to go to the washroom, indicating the caregiver can phone them with questions or, at a home visit, suggesting the caregiver accompany them to their car. One social worker went further in describing their approach to finding opportunities to initiate a conversation that can be conducive to assessing caregivers’ wellbeing and needs while simultaneously keeping patients’ preferences in mind:

*I would say to a patient: you know I'm going to be speaking with your caregiver, is that OK? And this is what I would like to be speaking with them about. Is there anything that you don't want me to talk to them about?* (Participant 77)

Other HCPs incorporate informal assessments of caregivers into their standard assessment of the patient by asking the caregiver questions similar to those asked of the patient, thereby including both the caregiver and the patient in the discussion.

## Discussion

Our findings highlight the contrasting preferences of having open versus private communication about caregiver challenges and caregiver-reported outcomes with the patient. Complementing these findings, HCPs described positive and negative aspects of open versus private communication, as well as contingent strategies they used to navigate sensitivities inherent in caregiver and patient preferences.

The fear of becoming a burden and desire to protect the other from difficult emotions has been articulated in research with cancer patients as well as caregivers [26]. According to Warren and Sakellariou [27], the distribution of caregiving roles is not unidirectional between the patient and the caregiver, such that emotion work is a mutual practice. That is, both the patient and the caregiver can work to alleviate the pressures that cancer places on the relationship [28]. A UK study of caregivers of CRC patients reported tension between the need for emotional expression and meaningful silences about their struggles [28]. Caregiver silence served to contain their suffering, protected the patient from difficult emotions, and facilitated caregivers in attending to patient needs and, thus, the caregiver’s relational commitments. Moreover, concealing their struggles helped maintain the idealized role of the selfless caregiver.

In our research, we found that while many caregivers and patients were aware of the mutual practice of tending to the emotions of the other, there were contrasting perspectives as to whether the patient should even be supporting the caregiver. Preferences for not sharing caregiver struggles and caregiver-reported outcomes with the patient can be interpreted as meaningful silences that enabled caregivers to fulfill their relational commitments and uphold the selfless caregiver ideal. Arteaga [28] challenged the ideas that the voicing of emotional distress is the only healthy way of coping, and that silence is a maladaptive coping strategy that represents a failure of caregiver-patient communication. Arteaga argued that concealing emotional struggles can be a form of productive emotion work that keeps lives going, with silence aiding healing and representing a moral choice to look after others, thus being a component of ordinary practices of care. In other words, choosing to share caregiver challenges is a relational practice of moral work [28]. Thus, the sharing of caregiver-reported outcomes with the patient in instances where this is not preferred by the caregiver or the patient could inadvertently undermine their functional manner of coping and, ultimately, their close relationship.

Our findings remind us that caregiving is more than an activity (caring for); it is a relationship wherein caring about a person reflects concern for, and feelings of responsibility for a person in need of care [29, 30]. It is these concerns that appear to influence whether the caregiver or the patient preferred openness or privacy. Further, our findings highlight that open and transparent communication about caregiver challenges between the caregiver and the patient were not preferable unless they had previously developed this type of communication pattern. Indeed, the HCPs in this study raised concern about causing harm to those in relationships where this was not usual. These findings suggest that the integration of caregiver assessments and formal caregiver-reported outcomes will require careful consideration of patient and caregiver preferences and, perhaps, their capacity to communicate about their challenges. Caregiver-reported outcome implementation would also benefit from careful consideration of available supports and interventions that target communication and interpersonal intimacy. Such interventions, most often dyad-based, provide coaching on how to communicate shared concerns and overcome the isolation or avoidance common as caregivers and patients attempt to protect each other from their own suffering [31]. After all, there is strong evidence that communication is important to facilitate improved individual psychological and physical health outcomes for cancer patients and their caregivers, as well as dyadic relationship-focused outcomes [2, 31].

Like other research [32, 33], some HCPs used informal and unstructured discussions to assess caregivers’ needs and concerns. However, many HCPs do not feel they have the time [32, 33] and/or the skills to have comprehensive discussions [34]. Thus, whether caregivers’ needs are assessed and identified, and to some extent addressed, becomes more a matter of chance than a system-level effort. A more structured approach to guide communication about caregivers’ needs could work as an intermediate step towards developing more standardized instruments and procedures for caregiver-reported outcome assessments.

The strengths of this research lie in the inclusion of caregiver, patient, and HCP participants, the diverse participant characteristics, and the depth of information obtained during interviews. Since the overwhelming majority of caregiver and patient participants chose an individual interview, we contend that we were able to obtain perspectives that participants might not have been comfortable voicing in front of each other. Of note, the large majority (22/25) of caregivers in our study identified as a woman, and thus, the findings perhaps under-represent the perspectives of caregivers of other genders. Though we attempted to recruit caregivers of diverse genders, we were unable to conduct purposive sampling due to COVID-19 related public-health measures that prevented in-clinic or in-person recruitment. Further, this research was conducted in Canada, via virtual interviews during the COVID-19 global pandemic, at a time when there were intermittent limits on in-person healthcare assessments, and a real or perceived lack of access to primary care, oncologic care, and home and social supports; these factors and this context may have impacted participant responses, and ought to consider when applying insights to other settings.

## Conclusion

It may be tempting to develop and implement caregiver-reported outcomes without considering the preferences of caregivers and patients themselves. However, the integration of formal caregiver-reported outcomes will require careful consideration of caregiver and patient preferences, with accompanying support and strategies, so as not to further caregiver burden. Clearly, supports and resources that complement caregiver-reported outcomes will be an important future research priority.

## Data Availability

The data that support the findings of this study are not available on request from the corresponding author nor are they publicly available because they contain information that could compromise the privacy of research participants.

## Abbreviations

CRC: Colorectal cancer
HCPs: Healthcare providers

## References

[1] Borsky AE, Zuvekas SH, Kent EE, de Moor JS, Ngo-Metzger Q, Soni A (2021). Understanding the characteristics of US cancer survivors with informal caregivers. Cancer.

[2] Northouse LL, Katapodi MC, Schafenacker AM, Weiss D (2012). The impact of caregiving on the psychological well-being of family caregivers and cancer patients. Semin Oncol Nurs.

[3] Zarit SH, Todd PA, Zarit JM (1986). Subjective burden of husbands and wives as caregivers: a longitudinal study. Gerontologist.

[4] Hunt G, Longacre M, Kent E, Weber-Raley L (2016). Cancer caregiving in the US: an intense, episodic, and challenging care experience. Natl Caregiv.

[5] Ochoa CY, Lunsford NB, Smith JL (2020). Impact of informal cancer caregiving across the cancer experience: a systematic literature review of quality of life. Palliat Support Care.

[6] Schulz R, Beach SR (1999). Caregiving as a risk factor for mortality: the Caregiver Health Effects Study. JAMA.

[7] Ferrell BR, Kate Kravitz R, Tami Borneman R (2018). Family caregivers. Clinic J Oncol Nurs.

[8] Girgis A, Lambert S, Johnson C, Waller A, Currow D (2012). Physical, psychosocial, relationship, and economic burden of caring for people with cancer: a review. J Oncol Pract.

[9] Howell D, Molloy S, Wilkinson K, Green E, Orchard K, Wang K (2015). Patient-reported outcomes in routine cancer clinical practice: a scoping review of use, impact on health outcomes, and implementation factors. Annals Oncol.

[10] Chen J, Ou L, Hollis SJ (2013). A systematic review of the impact of routine collection of patient reported outcome measures on patients, providers and health organisations in an oncologic setting. BMC Health Serv Res.

[11] Kotronoulas G, Kearney N, Maguire R, Harrow A, Di Domenico D, Croy S (2014). What is the value of the routine use of patient-reported outcome measures toward improvement of patient outcomes, processes of care, and health service outcomes in cancer care? A systematic review of controlled trials. J Clin Oncol.

[12] Marshall S, Haywood K, Fitzpatrick R (2006). Impact of patient-reported outcome measures on routine practice: a structured review. J Eval Clinic Pract.

[13] Yang LY, Manhas DS, Howard AF, Olson RA (2018). Patient-reported outcome use in oncology: a systematic review of the impact on patient-clinician communication. Support Care Cancer.

[14] Basch E, Deal AM, Dueck AC, Scher HI, Kris MG, Hudis C (2017). Overall survival results of a trial assessing patient-reported outcomes for symptom monitoring during routine cancer treatment. JAMA.

[15] Howell D, Li M, Sutradhar R, Gu S, Iqbal J, O’Brien MA (2020). Integration of patient-reported outcomes (PROs) for personalized symptom management in “real-world” oncology practices: A population-based cohort comparison study of impact on healthcare utilization. Support Care Cancer.

[16] Lambert SD, Brahim LO, Morrison M, Girgis A, Yaffe M, Belzile E (2019). Priorities for caregiver research in cancer care: an international Delphi survey of caregivers, clinicians, managers, and researchers. Support Care Cancer.

[17] Ahmed S, Barbera L, Bartlett S, Bebb D, Brundage M, Bryan S (2020). A catalyst for transforming health systems and person-centred care: Canadian national position statement on patient-reported outcomes. Curr Oncol.

[18] Klagholz SD, Ross A, Wehrlen L, Bedoya SZ, Wiener L, Bevans MF (2018). Assessing the feasibility of an electronic patient-reported outcome (ePRO) collection system in caregivers of cancer patients. Psycho-Oncol.

[19] Lambert-Obry V, Gouault-Laliberté A, Castonguay A, Zanotti G, Tran T, Mates M (2018). Real-world patient-and caregiver-reported outcomes in advanced breast cancer. Curr Oncol.

[20] Canadian Cancer Society (2019) Treatments for colorectal cancer. https://www.cancer.ca/en/cancer-information/cancer-type/colorectal/treatment/?region=on Accessed 12 Aug 12 2019.

[21] Thorne S (2016). Interpretive description: qualitative research for applied practice.

[22] Alberta SPOR SUPPORT Unit (2018) Patient engagement in health research: a how-to guide for researchers

[23] Howard AF, Lynch K, Beck S, Torrejón MJ, Avery J, Thorne S, Porcino A, De Vera M, Lambert L, Wolff A, McDonald M (2021) At the heart of it all: emotions of consequence for the conceptualization of caregiver-reported outcomes in the context of colorectal cancer. Current Oncol Oct (5):4184–202 10.3390/curroncol28050355 PMC8534905 34677273

[24] Malterud K, Siersma VD, Guassora AD (2016). Sample size in qualitative interview studies: guided by information power. Qual Health Res.

[25] Strauss A, Corbin J (1998) Basics of qualitative research: Techniques and procedures for developing grounded theory. Sage Publications, Inc

[26] Emslie C, Browne S, MacLeod U, Rozmovits L, Mitchell E, Ziebland S (2009). ‘Getting through’not ‘going under’: a qualitative study of gender and spousal support after diagnosis with colorectal cancer. Soc Sci Med.

[27] Warren N, Sakellariou D (2020). Neurodegeneration and the intersubjectivities of care.

[28] Arteaga Pérez I (2020). Emotion work during colorectal cancer treatments. Med Anthropol.

[29] Cash B, Hodgkin S, Warburton J (2013). Till death us do part? A critical analysis of obligation and choice for spousal caregivers. J Gerontol Soc Work.

[30] Glenn EN (2010). Forced to care: coercion and caregiving in America.

[31] Ferrell B, Wittenberg E (2017). A review of family caregiving intervention trials in oncology. CA Cancer J Clin.

[32] Riffin C, Wolff JL, Pillemer KA (2021). Assessing and addressing family caregivers' needs and risks in primary care. J Am Geriatr Soc.

[33] Parmar J, Anderson S, Abbasi M, Ahmadinejad S, Chan K, Charles L (2021). Family physician’s and primary care team’s perspectives on supporting family caregivers in primary care networks. Int J Environ Res Public Health.

[34] Koenig Kellas J, Castle KM, Johnson AZ, Cohen MZ (2021). Cancer as communal: Understanding communication and relationships from the perspectives of survivors, family caregivers, and health care providers. Health Commun.

